# Seroprevalence and characterisation of herpes simplex virus from human immunodeficiency virus in samples collected from the North-West and KwaZulu-Natal Provinces: a retrospective study

**DOI:** 10.12688/f1000research.28105.1

**Published:** 2021-02-11

**Authors:** Oluwafemi Samuel Obisesan, Nomathamsanqa Patricia Sithebe, Hazel Tumelo Mufhandu

**Affiliations:** 1 Department of Microbiology, North-West University, Mafikeng, South Africa

**Keywords:** Co-infection, Enzyme Linked Immunosorbent Assay, Herpes Simplex Virus, Human Immunodeficiency Virus, Polymerase Chain Reaction.

## Abstract

**Background:** Herpes simplex viruses (HSVs) are highly pervasive and show a strong synergistic interaction with human immunodeficiency virus (HIV). High prevalence of HSV type 1 (HSV-1) has been reported in Africa with a prevalence rate of 20-80% in women and 10-50% in men. Studies on the prevalence of HSV in South Africa are few considering the rate of HIV infection in the country. Our focus was to determine the molecular prevalence of HSV-DNA in HIV-1 sera.

**Methods:** In total, 44 convenience samples were screened for HSV and HIV-1 using the highly sensitive enzyme-linked immunosorbent assay (ELISA). The ELISA positive samples were characterized using polymerase chain reaction (PCR) to confirm the positivity of both viruses and to further differentiate HSV into HSV-1 and -2. Thereafter, the samples were analysed for relatedness using phylogenetic analysis.

**Results:** Of 44 samples, 36 (81.8%) were positive for HIV-1, while 35 (79.5%) were positive for HSV when screened with ELISA kits. The results of PCR with type specific primers showed that 4/35 (11.4%) samples were specific for HSV-1 while 30/35 (85.7%) were specific for HSV-2. Statistical analysis performed using chi-squared goodness-of-fit test showed that there is a significant relationship between HSV-2 and HIV-1 transmission.

**Conclusions:** High prevalence of HSV-2 recorded in HIV-1 sera corroborate with similar studies conducted within different cohorts in the continent. SPSS Pearson’s chi-squared test established that there is a significant relationship between HSV-2 and HIV-1 transmission.

## Introduction

Herpes simplex virus (HSV) is a prevalent organism that belongs to the sub-family of alpha Herpesviridae ( Greninger *et al*., 2018). The majority of HSV transmission is asymptomatic because carriers are not aware of their infection, a factor that is partly responsible for the high prevalence of HSV infection worldwide. On the other hand, HSV transmission can produce symptomatic lesions in the infected host ( Looker *et al*., 2015). When symptoms appear, there is an increase in the virus titre ( Mazzarello *et al*., 2018). Exposure to an infected person or cell fuels the transmission of the virus ( Jaishankar & Shukla, 2016). Transmission of this herpetic virus can be through oral or genital contact with the lesion. These routes of transmission berth HSV differentiation into two types, HSV-1 and HSV-2, respectively ( Looker *et al*., 2008; Schiffer *et al*., 2014). HSV-1 is the cause of blisters and sores around the mouth, while HSV-2 is traditionally associated with blisters around the genitals, although, both viruses can cause infection at both sites ( Grinde, 2013; Modi *et al*., 2008).

The prevalence of HSV-1 and HSV-2 infection in the United States from a survey conducted in 2018 was reported as 47.8% and 11.9%, respectively ( McQuillan *et al*., 2018). In Africa, the rate of occurrence of HSV-2 is 20–80% in women, 10–50% in men, while that of HSV-1 is 49.7% in women and 50.3% in men ( Looker *et al*., 2015; WHO, 2015). HSV-2 is the most common virus responsible for genital ulcer diseases ( Johnston & Corey, 2016). Feng *et al*. (2013) stated that genital ulcerations caused by these viruses is a burden globally with a worldwide estimate of >500,000,000 people (15% prevalence) infected with genital herpes between the ages of 15–49 years. In South Africa, a study conducted in KwaZulu-Natal by Rajagopal *et al.* (2014), showed a 84% incidence of HSV-2 among commercial sex workers.

Recently, HSV-1 has become a major factor influencing genital herpes in most industrialised nations with about 140 million people, aged 15–49 years, infected with genital HSV-1 ( WHO, 2015). The change in virus causing genital herpes is due to the downward shift in trend of HSV-1 acquisition before sexual relations. In addition, children who do not have HSV-1 antibodies at the early stage are vulnerable to genital HSV-1 infection when exposed ( Bradley *et al*., 2014; Forward & Lee, 2003).

According to UNAIDS (2019), HIV-1 prevalence in South Africa was estimated at 7.7 million in 2018 making it the highest epidemic in the world. Considering the high prevalence of human immunodeficiency virus (HIV) in South Africa and the role that HSV plays in its transmission, the need to determine the prevalence of HSV and HIV-1 co-infection is of great importance. HSV is an important co-factor in HIV acquisition due to physical disruption of the epithelial surface at the infection site. This serves as a port of entry for HIV recruitment and can facilitate its transmission by two to three fold ( Looker *et al*., 2017; Munawwar & Singh, 2016). HSV makes the transmission of HIV effortless by causing frequent replication of HSV; hence, causing disease progression. The effect of HSV in HIV acquisition has been observed in Western Asia, Europe and Africa where the prevalence of HSV-2 in HIV infected populations was 60–90%, 30–70% and 50–90%, respectively, which is three times the rate of infection in normal populations ( Anaedobe & Ajani, 2019; Des Jarlais *et al*., 2014; Mohraz *et al*., 2018).

It is apparent that a well-built interaction exists between HSV-2 and HIV-1 infection ( Barnabas & Celum, 2012; Kolawole *et al*., 2016; Todd *et al*., 2013), although there are few contrasting opinions. In 2006, Freeman and colleagues conducted a systematic review on gender-based effect of HSV-2 in the transmission of HIV infection ( Freeman *et al*., 2006). In their study, they discovered that HSV-2 is a significant facilitator for HIV transmission in men and women. In addition, there is a three-fold risk of HIV-1 acquisition in HSV-2 infected persons in sub-Saharan Africa ( Barnabas & Celum, 2012). Most research that recognises the association between HSV-2 and HIV-1 has been conducted outside South Africa. However, the incidence of HSV-2 and HIV-1 co-infection reported in South African women was 41% ( Abbai *et al*., 2015).

The focus of this study was to establish the prevalence of HSV antibodies and HSV-DNA in HIV-1 sera collected from National Health Laboratory Service (NHLS) laboratories. In addition, HSV-2 and HIV-1 co-infections were examined in the samples.

## Methods

### Sample collection criteria and study population

Samples from patients who attended a NHLS clinic in the North-West Province and KwaZulu-Natal Province for HIV screening and management were used. Convenience sampling was used. Patients had attended the laboratories with health conditions that were discrete from HSV infection. From the laboratories, 44 sera samples were collected over a period of one year (July 2017 – May 2018); 20 sera samples from North-West and 24 from KwaZulu-Natal Provinces. The samples were collected blindly from the two clinical laboratories with no additional data except for the age and sex of the participants. Sera were kept in sterile vials and stored at -80°C until they were ready for use.

### Laboratory analysis

Kasubi (2006) has stated that the majority of the screening on Africa sera samples are biased because of their high false positive results. In addition, Satpathy *et al*. (2018) has stated that “EIA and PCR are more reliable and accurate detection techniques for HSV and HIV-1”. These two statements were considered while selecting the laboratory technique to assess HSV. The statements informed our decision to use enzyme-linked immunosorbent assay (ELISA) and polymerase chain reaction (PCR) techniques to analyse the samples. ELISA was used to detect the presence of viral antibodies (HSV and HIV-1) in the sera samples and PCR was used to confirm the positivity of the samples and differentiate HSV samples into types 1 and 2. 

### ELISA

A highly sensitive but non-specific ELISA test kit, Platelia HSV (1+2) (Bio-Rad, Marnes-la-Coquette, Paris, France) and Genscreen Ultra HIV Ag-Ab test kit (Bio-Rad, Marnes-la-Coquette, Paris, France) were used to detect the presence of HSV-1 and -2 and HIV-1 antibodies, respectively. The two kits were used to test all 44 samples. The manufacturer’s instructions were strictly followed with little modifications where necessary, as reported in our previous work, Obisesan (2019).

### DNA isolation and PCR

DNA and RNA were extracted from the sera samples using QIAamp® MinElute® Virus Spin kit and QIAamp® Viral RNA Mini kit (Whitehead Scientific, Cape Town, South Africa), respectively, following the manufacturer’s protocols. The extracted material was aliquoted and stored at -80°C until further use.

PCR was run on each ELISA positive sample (HSV types 1 and 2 and HIV-1) using viral gene specific primers. The primers were selected based on previous studies ( Nie *et al.,* 2011; Schmutzhard *et al.,* 2004; Victória *et al.,* 2005), for the detection of HSV-1, HSV-2 and HIV-1, respectively, as outlined in the *Extended data* (Table A). Briefly, 25 µl PCR reaction mixture of Quick-Load® *Taq* 2X Master Mix kit (Biolabs) with each primer set targeting the different regions was prepared ( *Extended data*, Table B) and amplification was performed on a T100 ^TM^ thermal cycler (Bio-Rad, Hercules, California, United States). HSV-1 and -2 amplification was completed with primer annealing temperature at 55°C for first round PCR and 61.4°C for nested PCR, with a total of 35 cycles for both, as outlined in the *Extended data* (Table B). A two-step reverse transcription PCR was used to amplify the HIV-1 gene of interest from the extracted RNA material. This was achieved with a primer annealing temperature of 50°C for 30 cycles, as outlined in the *Extended data* (Table C). The PCR products were electrophoresed on a 2% agarose gel, stained with ethidium bromide and identified by UV light transilluminator (MUV26 Series, Major Science, California, USA).

### DNA sequencing

Next-generation sequencing (NGS) was used to validate the detected viral genomes of the clinical samples. Only the samples that exhibited high titers of the two individual viruses (HSV-2 and HIV-1) were processed through to sequencing. DNA sequencing was performed on four HSV-2/HIV-1 co-infected samples with the highest titre as identified with ELISA. The samples were annotated as G13, G15 and G34, and G20. HSV-2 primers were used to sequence G13, G15 and G34 while the G20 sequencing was done using HIV-1 primers. The next generation sequencing was carried out on the Illumina MiSeq NGS platform at Inqaba Biotec (Inqaba Biotechnical Industries (Pty) Ltd, Johannesburg, SA). The phylogenetic analysis was performed by trimming and aligning the sequences using BowTie 2 v 2.3.2 ( Langmead & Salzberg, 2012). All aligned data were further annotated to determine the viral genome using Prokka v 1.12 ( Seemann, 2014). The sequence data, after trimming and annotation, was subjected to Molecular Evolutionary Genetics Analysis (MEGA 7) against HIV-1 and HSV-2 reference genomes (see Results) obtained from National Centre for Biotechnology Information (NCBI).

### Statistical analysis

Data analyses were carried out on Statistical Package of Social Sciences (SPSS) software (version 25). The chi-squared goodness-of-fit test (x ^2^) was used to evaluate for an association between the categorical variables. Relationship between the demographics and the viruses was tested using Pearson correlation coefficient. The 5% significance level was considered as significant p value in this study

### Ethical approval

The study received ethical approval from the North-West University Research Ethics Regulatory Committee (NWU-00068-15-A9) prior to the study. Also, we received permissions from NHLS to make use of the samples of those patients who gave their verbal consent for use of their samples in the study.

## Results

The demographics of the study population showed that majority of the study participants were female (79.5%) and a lesser percentage (20.5%) of the population were male ( Table 1). The sample size is considered as one of the study limitations. The mean age and standard deviation of the study population were 33.09 ±11.94 years.

**Table 1.** Demographics and ELISA screening results of HSV and HIV-1 sera.

Age Group	HIV-1 Positive		HIV-1 Negative		HSV Positive		HSV Negative	
	Male	Female	Male	Female	Male	Female	Male	Female
<10	0	0	0	0	0	0	0	0
11–20	0	5	1	2	0	5	1	2
21–30	4	5	1	2	4	4	1	2
31–40	1	9	0	0	1	8	0	2
41–50	1	9	0	2	1	10	0	1
51–60	1	1	0	0	1	1	0	0
**TOTAL**	7	29	2	6	7	28	2	7

**Figure 1.**
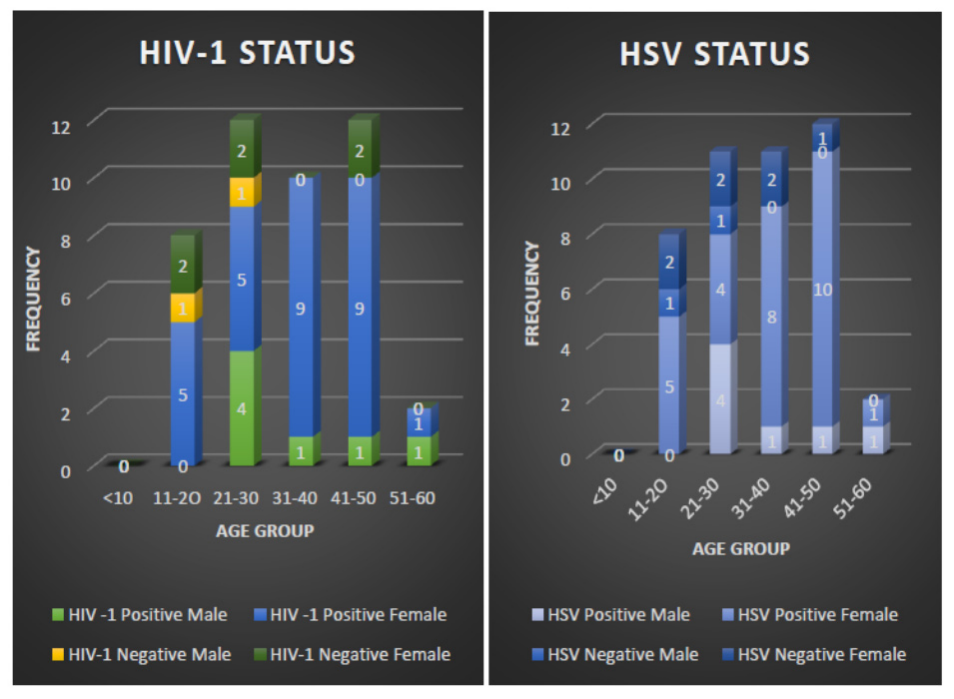
ELISA screening and gender categorisation of HSV and HIV-1 within the study population.

ELISA screening of the samples showed that 36/44 (81.8%) were seropositive for HIV-1 ( Figure 1 and *Extended data*, Figure A) while 35/44 (79.5%) were positive for either HSV-1/2 antibody ( Figure 1). Notably, the study participants within the age group of 41–50 years had the highest HSV and HIV-1 infection rates, as depicted in Figure 1.

The amplification of glycoprotein B region of HSV using PCR showed that 4/35 (11.4%) of the HSV positive samples tested positive for HSV-1. Similarly, in order to differentiate the HSV positive samples into HSV-2, PCR amplification of glycoprotein G region of HSV positive samples was done. The result showed that 30/35 (85.7%) were HSV-2 positive. The gender distribution of these viruses in the study participants are shown in the *Extended data* (Figure B and C) for HSV-1 and HSV-2, respectively. Another finding from the study was that 1/44 (2.3%) males and 2/44 (4.5% ) females from the study population were HSV-1/HIV-1 co-infected. Furthermore, a significant proportion of the population, 6/44 (13.6%) males and 24/44 (54.5%) females were HSV-2/HIV-1 co-infected.

NGS was performed to detect and confirm the genomes of the viral isolates (HSV-2 and HIV-1) from the clinical samples. The sequenced data was compared with reference genomes of HSV-2 and HIV-1 obtained from NCBI. The reference genomes were selected based on the amplified targeted regions, that is, glycoprotein G for HSV-2 and integrase for HIV-1. The selected HSV-2 reference genomes are HSV-2 SD90e ( KF781518), HSV-2 strain 333 ( M15118) and glycoprotein G-2 ( AF141858); and for HIV-1 they are HIV-1 LC022388, HIV-1 LC201873, HIV-1 isolate 3792/15 KU609388 and HIV-1 isolate KU609428.

**Figure 2.**
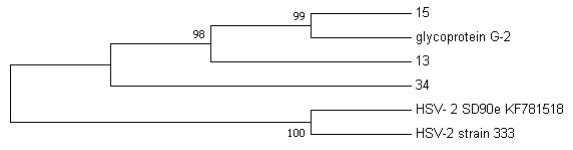
The evolutionary relationship between HSV-2 sequences (13, 15 and 34) and HSV-2 reference genomes (HSV-2 strain 333, HSV-2 SD90e and glycoprotein G-2, from NCBI database) was inferred using maximum likelihood method.

**Figure 3.**
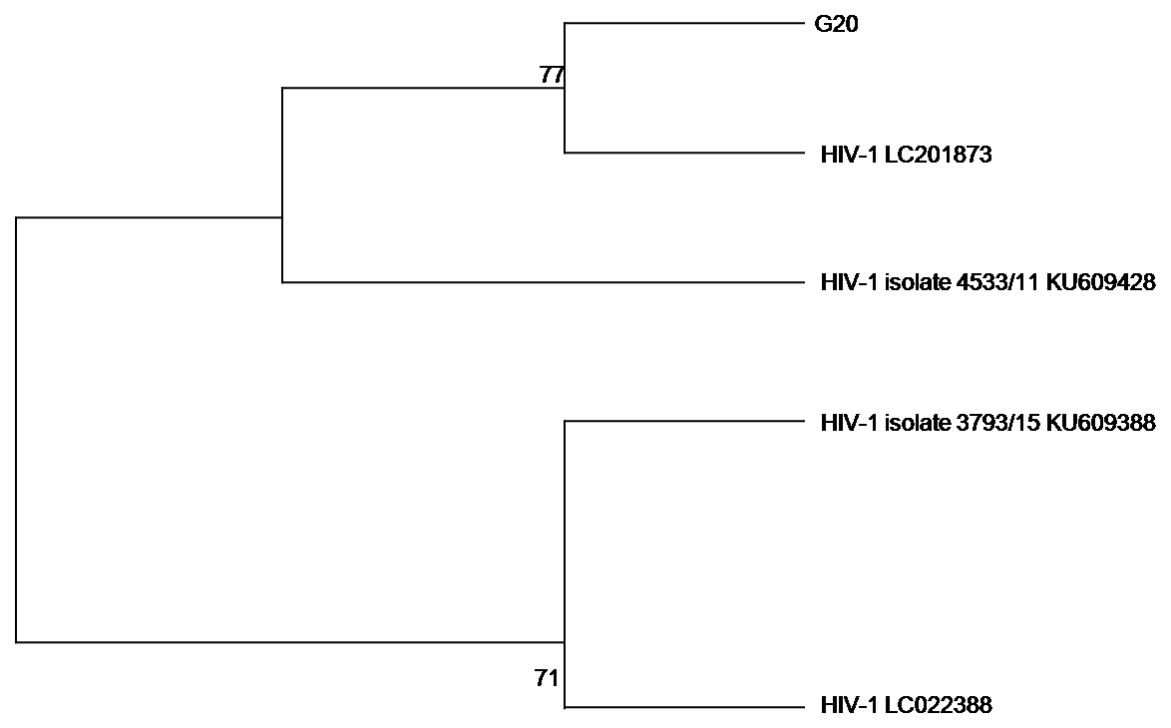
The evolutionary relationship of the HIV-1 sample (G20) with HIV-1 reference genomes from NCBI database was inferred using maximum likelihood method.

Phylogenetic analysis was performed on the sequence data to check the evolutionary relatedness of the sequenced data and the selected reference genomes. We decided to use a maximum likelihood method of analysis with a bootstrap phylogeny method (1,000 bootstrap replicates), that generated evolutionary trees as illustrated in Figure 2 and Figure 3 for HSV-2 and HIV-1, respectively.

The HSV-2 phylogeny tree ( Figure 2) clearly showed that nucleotide sequence 13 is closely related to sequence 15 more than sequence 34, with 98–99% similarity to glycoprotein G2 reference strain.

The HIV-1 phylogeny tree in Figure 3 showed that the G20 sequence is 77% closely related to HIV LC201873 and HIV-1 isolate 4533/11 KU609428; and distantly related to the other HIV-1 reference genomes (HIV-1 LC022388 and HIV-1 isolate 3793/15 KU609388) used in constructing the phylogenetic tree.

SPSS v25 was used to establish that a possible relationship exists between age, HSV, and HIV-1. SPSS was also utilized to evaluate the association between HSV and HIV-1 positive samples. The two-tailed correlation test exhibited a statistical link between age and HSV-1 (0.366**), HSV-2 and HIV-1 samples (0.690**) and an inverse relationship between HSV-1 and HSV-2 samples (-0.463**), as shown in Table 2.

**Table 2.** Correlation between age, HIV-1 ELISA positive samples, HSV-1 and HSV-2 PCR positive samples.

	AGE	HSV-1	HSV-2	HIV-1
**AGE**	1	0.366 **	-0.061	0.208
**HSV-1**	–	1	-0.463 **	-0.056
**HSV-2**	–	–	1	0.690 **
**HIV-1**	–	–	–	1

**Correlation is significant at 0.01 level (2-tailed)

The SPSS chi-square goodness-of-fit test was also used to assess association between HSV positive samples and HIV-1 sera. The data showed that there was no significant association between HSV-1 and HIV-1 (X ^2 ^(1) = 0.138, p>0.05). However, there was a strong statistical association between HSV-2 and HIV-1 (X ^2 ^(1) = 20.952, p< 0.05), as shown in Table 3.

**Table 3.** Association between HIV-1 ELISA positive samples and HSV-1 and HSV-2 PCR positive samples.

	HIV-1 ELISA			
	X ^2^ Value	Degree of freedom (df)	Asymptotic Significance (2-sided)	Interpretation
**HSV-1 PCR**	0.138	1	0.711	No significant association
**HSV-2 PCR**	20.952	1	0.000 *	Significant association

*Significant at 0.05 level (2-tailed).

## Discussion

There are insufficient data to recount the prevalence of HSV genotypes among HIV-1 infected individuals in the Republic of South Africa. This study provides insight into an existing interrelation between HSV and HIV-1 and the potential risk that one of the viruses may have on the other. Our focus was to determine the seroprevalence of HSV among HIV-1 sample cohort. The study also assessed for possible co-infections (HSV-1/HIV-1 or HSV-2/HIV-1). The study detected 81.8% HIV-1 and 79.5% HSV positive samples, with 85.7% of the HSV samples being positive for HSV-2. Conversely, HSV-1, which is the most prevalent type of HSV, as recorded by previous studies, was not highly prevalent in this study population (11.4%). The study also revealed a higher co-infection rate of HSV-2/HIV-1 (13.6% males, 54.5% females) compared with HSV-1/HIV-1 (2.3% males, 4.5% females).

One of the few trends drawn from this study was that the prevalence of HSV-2 as compared to HSV-1 in HIV-1 co-infection was relatively high. The validity of the high prevalence of HSV-2/HIV-1 co-infection in this study is further supported by previous studies ( Looker *et al.,* 2017; Patel *et al.,* 2012). Furthermore, it was discovered that females were more susceptible to HSV infection 30/35 (85.7%) than their male counterparts 4/35 (11.4%) in this population. This auspiciously matches with findings of Pebody *et al.* (2004) and Smith & Robinson (2002), who reported that women are more at risk of acquiring HSV-2 infection compared with men. This was also observed by the findings of Celum *et al*. (2004), that more than half of the female population who are HIV-1 positive suffer from HSV-2 infection. This might be due to their early exposure to sexual relations than their male counterpart ( Schiffer *et al.*, 2014). Moreover, the highest number of HSV and HIV-1 prevalence was observed in the age group of 41–50 years. However, only 3/44 (6.8%) HSV-1/HIV-1 co-infected samples were recorded in this study. Of note is that the rate of co-infection increased with age, which correlates with the steady rise by age for HSV-2/HIV-1 co-infection, as recorded by Beydoun *et al.* (2010).

PCR was performed to confirm the positivity of the ELISA screened samples and differentiate the HSV samples into types 1 and 2. The results (11.4% HSV-1 and 85.7% HSV-2) correlate with the high rate of HSV-2 infection in Africa. HSV-2 infection rates were previously reported in Zimbabwe (68%), Uganda (58%), Zambia (55%) and Gambia (28%) ( Debrah *et al*., 2018; Looker *et al*., 2015; Paz-Bailey *et al*., 2007). The decline in the prevalence rate of HSV-1 in this study is in contrast with the high HSV-1 prevalence observed by Cunningham *et al.* (2006) with 80% in women and 71% in men. This decline was also stated by Ayoub *et al*. (2019), who added that more children will reach the age of sexual debut with no antibody protection against HSV-1. In addition, the decline is attributable to the change in disease spread in the current population owing to the fact that there are reduced viral HSV-1 antibodies at a very young age, a factor influencing HSV-2 acquisition.

Phylogenetic analysis revealed that HSV-2 samples 13 and 15 from this study do not share the same ancestral lineage with a more virulent clinical isolate SD90e (accession number KF781518) and the HSV-2 laboratory strain 333 (M15118). A previous study by Newman *et al*. (2015) showed that SD90e viral sequence originated from South Africa. However, the distant relation between SD90e reference genome and samples 13 and 15 may suggest geographical diversity in viral transmission ( Szpara *et al*., 2014). Furthermore, a close relation of the two samples (G13 and G15) was observed with the less virulent glycoprotein G-2 strain originating from Scotland, which is attributed to international migration. A study with a larger sample cohort is encouraged to confirm this claim.

The HIV-1 phylogenetic analysis demonstrated that there is a close relationship between the G20 sequence and HIV-1 LC201873, a resistant integrase strand transfer inhibitor (INSTI) strain found in Japan and the HIV-1 isolate 4533/11 KU609428 from Morocco. This may be a reflection of another instance of international migration ( Lurie *et al*., 2003). The three sequences were observed to be distantly related to HIV-1 isolate 3792/15 KU609388 and HIV-1 LC022388, both from Morocco.

A previous study on the relationship between age and HSV-1 prevalence by Smith & Robinson (2002) reports that global HSV-1 prevalence increases with age. The statistical relationship between age, HSV and HIV-1 infection in our study revealed a similar significant relationship between age and HSV-1 (p = 0.366*). In the same vein, we discovered a discreet relationship between age, HSV-2 and HIV-1. HSV-1 showed an inverse correlation with HSV-2 (p = -0.463 ^**^). This suggests that an increase in HSV-2 prevalence in the population will result in a drastic decline of HSV-1. It was also discovered that a robust significant relationship exists between HSV-2 and HIV-1 (p =0.690 ^**^) suggesting that a steady rise in HSV-2 leads to a rise in HIV-1 infection in the population. A probable reason for this relationship is that the viruses share a similar route of entry and the impact of one is significant on the other as seen in the micro-ulceration of the genitalia in HSV-2 patients, which provides a port of entry for HIV-1 ( Sheth *et al*., 2008).

The association between HIV-1 and HSV was analysed using chi-square goodness-of-fit test and it was discovered that no significant association exist between HSV-1 and HIV-1 (X ^2 ^(1) = 0.138, p >0.05) but a strong statistical association was found between HSV-2 and HIV-1 (X ^2 ^(1) = 20.952, p <0.05). Although, there has been contrasting opinions on the association of HSV-2 and HIV-1, the current study is consistent with Sudenga *et al.* (2012) and Omori & Abu-Raddad (2017) who suggested that the infection of one virus may fuel the transmission of the other. In their study, Sudenga *et al.* (2012) observed that HIV-1 positive individuals with higher CD4+ counts at baseline and those with lower viral load were associated with HSV-2 acquisition, while Omori & Abu-Raddad (2017) used the sexual network determinants as components to determine the prevalence of HIV/ HSV-2. However, they deduced that HIV is an agent of HSV-2 transmission in the population. The data from this study suggests that contracting one virus (either HSV-2 or HIV-1) will influence the acquisition of the other. A recent study led by Kouyoumjian *et al.* (2018) and his group discovered a robust association between HSV-2 and HIV-1, with HSV-2 prevalence being consistently higher than that of HIV-1 in the global population. This accentuates the importance for data collation on HSV-2 and HIV-1 infected persons in South Africa

### Limitations

This study is a retrospective study with the aim to determine the prevalence of HSV-DNA in HIV-1 sera. The study contributed widely to the literature by exploring a broad group (participants between 14 to 49 years) within a small population, but there were certain limitations. First, the study population was small when compared to the size of the general population. Second, the samples were collected exclusively from North-West and KwaZulu-Natal Provinces, hence, it is difficult to generalize the outcomes of the study to the South African populace.

## Conclusion

This study showed that HSV is highly prevalent with women being largely affected. Moreover, HSV-2 infection in the study cohort was robustly associated with HIV-1. This suggests that if HSV-2 infection is not properly managed in a setting with generalized HIV epidemic, it may add a burden to the already deteriorating health status of the affected persons leading to high mortality and morbidity. To acknowledge that a relationship exists between these two viruses, and to identify how the transmission of one could affect the other, requires a large cohort at risk for HIV that is well described with longitudinal measurements of HSV-1, HSV-2 and HIV-1 as well as measurements of potential confounders such as condom use, partner change and other sexually transmitted diseases. This will aid in reducing the rate of disease recurrence in the population and possibly prevent impending co-infections. In spite of the study limitations, the data adds to the body of knowledge on HSV infection and its co-infection with HIV-1.

## Data availability

### Underlying data

SRA: gG sequencing of HSV-2 (sample G15), Accession number SRX9590097: https://www.ncbi.nlm.nih.gov/sra/?term=SRX9590097

SRA: gG sequencing of HSV-2 (sample G34), Accession number SRX9590139: https://www.ncbi.nlm.nih.gov/sra/?term=SRX9590139

SRA: gG sequence (sample G13), Accession number SRX9590098: https://www.ncbi.nlm.nih.gov/sra/?term=SRX9590098

SRA: HIV-1 sequencing targeting integrase region (sample G20), Accession number SRX9531105: https://www.ncbi.nlm.nih.gov/sra/?term=SRX9531105

Dryad: HSV AND HIV RAW DATA, https://doi.org/10.5061/dryad.zs7h44j7g ( Obisesan *et al.,* 2020).

This project contains the following underlying data:

- CSV spreadsheet containing demographics and ELISA and PCR results for all 44 sera samples.

### Extended data

Dryad: F1000 SUPPLEMENTARY FILE, https://doi.org/10.5061/dryad.zs7h44j7g ( Obisesan *et al.,* 2020).

This project contains the following extended data:

- Table A: Primers for the detection of HSV-1, HSV-2 and HIV-1.
- Table B: PCR reaction mixture and thermocycling conditions for HSV-1 and -2.
- Table C: Thermocycling conditions for HIV-1 PCR.
- Figure A: ELISA results of HSV as recorded on the microplate reader.
- Figure B: Age variation and the frequency distribution of HSV-1 in the study population
- Figure C: Age variation and the frequency distribution of HSV-2 in the study population

Data are available under the terms of the Creative Commons Zero "No rights reserved" data waiver (CC0 1.0 Public domain dedication). 
