# Seroprevalence and characterisation of herpes simplex virus from human immunodeficiency virus in samples collected from two provinces in South Africa: a retrospective study

**DOI:** 10.12688/f1000research.28105.4

**Published:** 2021-11-17

**Authors:** Oluwafemi Samuel Obisesan, Nomathamsanqa Patricia Sithebe, Hazel Tumelo Mufhandu

**Affiliations:** 1 Department of Microbiology, North-West University, Mafikeng, South Africa

**Keywords:** Co-infection, Enzyme Linked Immunosorbent Assay, Herpes Simplex Virus, Human Immunodeficiency Virus, Polymerase Chain Reaction.

## Abstract

**Background: **Herpes simplex virus (HSV) is a widely distributed human pathogen that is known for its ulcerative lesions at the infection site. HSV can cause persistent infection in the host that is often followed by a period of latency within the neurons. Considering the high rate of HIV infection in South Africa, it is important to assess the seroprevalence of HSV with a focus to determine the epidemiological association between HSV-DNA and HIV-1 in the population.

**Methods: **A total of 44 sera samples were screened for HSV and HIV-1 using the highly sensitive enzyme-linked immunosorbent assay (ELISA). The ELISA positive samples were characterized using polymerase chain reaction (PCR) to confirm the positivity of both viruses and to further differentiate HSV into HSV-1 and -2. Thereafter, the samples were analysed for relatedness using phylogenetic analysis.

**Results: **Of the 44 samples, 36 (81.8%) were positive for HIV-1, while 35 (79.5%) were positive for HSV when screened with ELISA kits. The PCR results, with the use of type specific primers, showed that 4/35 (11.4%) samples were specific for HSV-1 while 30/35 (85.7%) were specific for HSV-2. Statistical analysis performed using the chi-squared goodness-of-fit test showed that there is a significant relationship between HSV-2 and HIV-1 transmission.

**Conclusions: **There is a significant positive association between HSV-2 and HIV-1 in the study population. Our study shows that some of the HSV-2 isolates are not related to the clinical isolate SD90e from South Africa, suggesting diversity in HSV-2 viral transmission.

## Introduction

Herpes simplex virus (HSV) is a prevalent organism that belongs to the sub-family of alpha Herpesviridae ( Greninger *et al*., 2018). The virus is transmitted either through oral or genital route. The routes of transmission of HSV are responsible for its differentiation into two types; herpes simplex virus types 1 (HSV-1) and 2 (HSV-2) ( Looker *et al*., 2008; Schiffer *et al*., 2014). HSV-1 is transmitted orally and it is the cause of blisters and sores around the mouth, while HSV-2 is sexually transmitted and it is traditionally associated with blisters or ulcers around the genitals ( Grinde, 2013; Modi *et al*., 2008). Conversely, most HSV infections are asymptomatic, a factor that is partly responsible for the high prevalence of HSV infection worldwide. However, some HSV infections show visible signs of infection in the host ( Looker *et al*., 2015), which is often associated with the increase in virus titer that further fuels its transmission ( Jaishankar & Shukla, 2016; Mazzarello *et al*., 2018).

It is important to determine the epidemiology of HSV-1 and HSV-2 because of the disease burden associated with their infection ( Chemaitelly *et al*., 2019). The global prevalence for HSV-1 and HSV-2 is approximately 3.7 billion and 500 million respectively ( Feng *et al*., 2013; WHO, 2015). In 2018, McQuillan and colleagues conducted a survey in the United States and estimated the prevalence of HSV-1 and HSV-2 as 47.8% and 11.9% respectively ( McQuillan *et al*., 2018). However, Africa has a higher rate of HSV-2 prevalence (20–80% and 49.7%) and HSV-1 (10–50% and 50.3%) in women and men, respectively ( Looker *et al*., 2015; WHO, 2015). HSV-2 is the most common virus responsible for genital ulcer diseases (GUD) ( Johnston & Corey, 2016); however, research has shown that GUD may also be caused by HSV-1 particularly in the industrialized nations ( WHO, 2015). The sudden increase in GUD caused by HSV-1 is attributable to the downward shift in trend of HSV-1 acquisition before sexual relations in this population. In addition, children who do not have HSV-1 antibodies in the early stages of life are vulnerable to genital HSV-1 infection when exposed ( Bradley *et al*., 2014; Forward & Lee, 2003). Daniels *et al*. (2016), evaluated the incidence of HSV-2 and its risk factors within a cohort of HIV-1 negative women in KwaZulu-Natal, South Africa. They observed that 84% female commercial sex workers in the study population were infected with HSV-2.

The high prevalence of human immunodeficiency virus type 1 (HIV-1) in South Africa (about 7.7 million) has made the Joint United Nations Programme on HIV/AIDS (UNAIDS) to regard the country as the epicenter of HIV-1 in the world ( UNAIDS, 2019). Considering the high prevalence of HIV-1 in South Africa and the role that HSV plays in its transmission, the need to determine the prevalence of HSV and HIV-1 co-infection is of great importance. In addition, HSV disrupts the epithelial surface at the infection site, which serves as a port of entry for HIV recruitment that progressively facilitates its transmission by two to three fold. This characteristic makes HSV an important co-factor in HIV acquisition ( Looker *et al*., 2017; Munawwar & Singh, 2016). HSV infection makes the transmission of HIV effortless through a transmission that disrupts the epithelium. Moreover, dual infection with HIV increases the rate of HIV replication, which further suppresses the immune system, thus enhancing disease progression. The effect of HSV in HIV acquisition has been observed in Western Asia, Europe and Africa where the prevalence of HSV-2 in HIV infected populations was 60–90%, 30–70% and 50–90%, respectively, which is three times the rate of infection in normal populations ( Anaedobe & Ajani, 2019; Des Jarlais *et al*., 2014; Mohraz *et al*., 2018).

It is apparent that a sturdy interaction exists between HSV-2 and HIV-1 infection ( Barnabas & Celum, 2012; Kolawole *et al*., 2016; Todd *et al*., 2013), although, a contrasting opinions was reported that HSV-2 co-infection with HIV has no role in increasing the transmission of HIV ( Mohraz *et al*., 2018). However, Freeman and colleagues conducted a systematic review on the gender-based effect of HSV-2 in the transmission of HIV infection ( Freeman *et al*., 2006). In their study, they discovered that HSV-2 is a significant facilitator of HIV transmission in both men and women. Another study reported a three-fold risk of HIV-1 acquisition in HSV-2 infected persons in the sub-Saharan Africa ( Barnabas & Celum, 2012). Most studies that discovered the association between HSV-2 and HIV-1 were conducted outside of South Africa. Albeit, one study reported an incidence of 41% of the co-infection in South African women ( Abbai *et al*., 2015).

Accordingly, the current study aims to establish the prevalence of HSV antibodies and HSV-DNA in HIV-1 sera and further assess the evidence of HSV-2 and HIV-1 co-infection within the study cohort.

## Methods

### Sample collection criteria and study population

The sera samples that were used in the study were previously stored sera collected from patients who visited Bophelong Provincial Hospital in Mafikeng, North-West Province, and Inkosi Albert Luthuli Central Hospital (IALCH-NHLS) in KwaZulu-Natal Province, both in South Africa. The study participants visited the hospitals for HIV screening and management in their respective provinces. Only 25 sera samples from each hospital (50) were selected for use in the current study. However, the sample quality (serum lysis) of six sera from the total samples prevented us from using them and were excluded, reducing the total sample number to forty-four (44). The samples were anonymized with no additional data except age and gender of the study participants.

### Laboratory analysis

The enzyme-linked immunosorbent assay (ELISA) was used to detect the presence of HSV and HIV-1 antibodies in the sera. Polymerase chain reaction (PCR) was used to confirm that the samples were infected with HSV and HIV-1 and to differentiate the HSV samples into HSV type 1 and 2.

### ELISA

A highly sensitive ELISA test kit for HSV, Platelia HSV (1+2) (Bio-Rad, Marnes-la-Coquette, Paris, France) was used to measure HSV IgG antibody in the samples. The Genscreen Ultra HIV Ag-Ab test kit (Bio-Rad, Marnes-la-Coquette, Paris, France) was used to detect HIV-1 p24 antigen in the sera. The ELISA plate was pre-coated with biotinylated polyclonal antibody to p24 HIV-1 Ag. The manufacturer’s instructions were followed with slight modifications. Briefly, the sera were diluted in a ratio 1:10 and 100 µl of the diluted sera, blank, positive, and negative controls were added to a flat-bottom 96-well plate. The plate was incubated at 37°C for 45 min and washed four times. Thereafter, the plate was incubated at room temperature for 15 min with 100 µl of substrate followed by 100 µl of stop solution (0.3M of H _2_SO _4_, Bio-Rad) to terminate the reaction. The plate was read on a microplate reader at 450 nm. The samples were run in duplicate and were considered positive for HSV IgG antibody if the ratio of the average of serum OD and the cut-off value was greater than 1.2, and negative if the ratio was less than 0.8.

### DNA isolation and PCR

DNA and RNA were extracted from the sera samples using QIAamp® MinElute® Virus Spin kit and QIAamp® Viral RNA Mini kit (Whitehead Scientific, Cape Town, South Africa), respectively, following the manufacturer’s protocols. 

PCR was performed using the samples that were positive for ELISA (HSV type 1 and 2 and HIV-1) with viral gene specific primers. Previously published primers from Nie *et al.* (2011); Victória *et al.* (2005) and Schmutzhard *et al.* (2004) were used to amplify the integrase, glycoprotein B (gB) and glycoprotein G (gG) region of HIV-1, HSV-1 and HSV-2 respectively, as outlined in the *Extended data* (Table A). The HSV positive samples were also tested for HIV-1 co-infection using PCR. This allowed the detection of HIV-1 and HSV-1 and/or -2 co-infections from the study samples. Briefly, 25 µl PCR reaction mixture (Quick-Load® *Taq* 2X Master Mix kit, Biolabs) with each primer set targeting the different regions, was prepared ( *Extended data*, Table B) and amplification was performed with the use of a T100 ^TM^ thermal cycler (Bio-Rad, Hercules, California, United States). HSV-1 and -2 amplification was performed with the nested PCR cycling conditions outlined in the *Extended data* (Table B). A reverse transcription PCR was used to amplify the HIV-1 integrase gene from the extracted HIV-1 RNA samples. This was achieved with the PCR cycling conditions outlined in the *Extended data* (Table C) and the PCR products were analysed by gel electrophoreses.

### DNA sequencing

Next-generation sequencing (NGS) was used to validate the genomes of the samples used. Only the samples that exhibited high titers of HSV-2 and HIV-1 with ELISA were sequenced. Thus, DNA sequencing was performed on four HSV-2/HIV-1 co-infected samples with the highest titers. The samples were annotated as G13, G15, G20 and G34. HSV-2 primers were used to sequence G13, G15 and G34 while the G20 sample was sequenced using HIV-1 primers since this was the only sample with a high HIV-1 titer. NGS was carried out on the Illumina MiSeq NGS platform at Inqaba Biotec (Inqaba Biotechnical Industries (Pty) Ltd, Johannesburg, SA). Phylogenetic analysis was performed by trimming and aligning the sequences using BowTie 2 v 2.3.2 ( Langmead & Salzberg, 2012). All aligned data were further annotated to determine the viral genome using Prokka v 1.12 ( Seemann, 2014). Thereafter, the sequence data were subjected to Molecular Evolutionary Genetics Analysis (MEGA 7) against HIV-1 and HSV-2 reference genomes obtained from National Centre for Biotechnology Information (NCBI). Phylogenetic analysis was performed to assess the evolutionary relatedness of the sequenced data in relation to the reference genomes. The HSV-2 published reference sequences that were used in the analysis were SD90e ( KF781518) from South Africa, HSV-2 strain 333 ( M15118), a wild-type laboratory reference strain from USA, and glycoprotein G-2 (AF141858), a European HSV-2 isolate. The HIV-1 reference sequences that were used in the analysis were two HIV-1 subtype C sequences from South Africa (HM569277 and HM569273), two HIV-1 subtype B from USA (AF203332) and Japan (LC022388), and HIV-1 subtype A (HM466997) from Europe. Thus, the reference sequences were selected to explore the relatedness of the sequenced samples with published reference strains from the country (South Africa) and from other developed countries. In addition, different HIV-1 subtypes were selected from different regions to identify the sub-type of our clinical isolate.

### Statistical analysis

Data analyses were carried out on Statistical Package of Social Sciences (SPSS) software (version 25). The chi-squared goodness-of-fit test (x ^2^) was used to evaluate for an association between the categorical variables. Relationship between the demographics and the viruses was tested using Pearson correlation coefficient. The 5% significance level was considered as a significant *p* value in this study.

### Ethical approval

The study received ethical approval from the North-West University Research Ethics Regulatory Committee (NWU-00068-15-A9).

## Results

The demographics of the study population showed that majority of the study participants were female (79.5%) with a low percentage of males (20.5%) ( Table 1). The mean age and standard deviation of the study population were 33.09 ±11.94 years.

**Table 1.** Demographics and ELISA screening of HSV IgG and HIV-1 p24 with a reflection of the demographics of the samples used in the study.

AGE GROUP	HIV-1 Positive		HIV-1 Negative		HSV Positive		HSV Negative	
	Male	Female	Male	Female	Male	Female	Male	Female
<20	0	5	1	2	0	5	1	2
21–40	5	14	1	2	5	12	1	4
41–60	2	10	0	2	2	11	0	1
**TOTAL**	**7**	**29**	**2**	**6**	**7**	**28**	**2**	**7**

ELISA screening of the samples showed that 36/44 (81.8%) were seropositive for HIV-1 while 35/44 (79.5%) were positive for either HSV-1/2 antibody ( Table 1 and *Extended data*, Figure A). Notably, the study participants within the age group of 21–40 years had the highest HSV and HIV-1 infection rates, as depicted in Table 1.

The PCR amplification of glycoprotein B region of HSV showed that 4/35 (11.4%) of the HSV positive samples were positive for HSV-1 ( Table 2 and *Extended data*, Figure B). Similarly, to differentiate the HSV-1 and HSV-2 positive samples, PCR amplification of the glycoprotein G region of HSV positive samples was performed ( Table 2). The data showed that 30/35 (85.7%) were HSV-2 positive [ Table 2 and Extended data (Figure C)]. Another finding was that 1/44 (2.3%) males and 2/44 (4.5%) females were HSV-1/HIV-1 co-infected.

**Table 2.** Gender distribution of HSV-1 and HSV-2 positive sera using polymerase chain reaction technique.

AGE GROUP	HSV-1 PCR Positive		HSV-2 PCR Positive	
	MALE	FEMALE	MALE	FEMALE
<20	0	0	0	5
21–40	0	1	5	11
41–60	1	2	1	8

**Figure 1.**
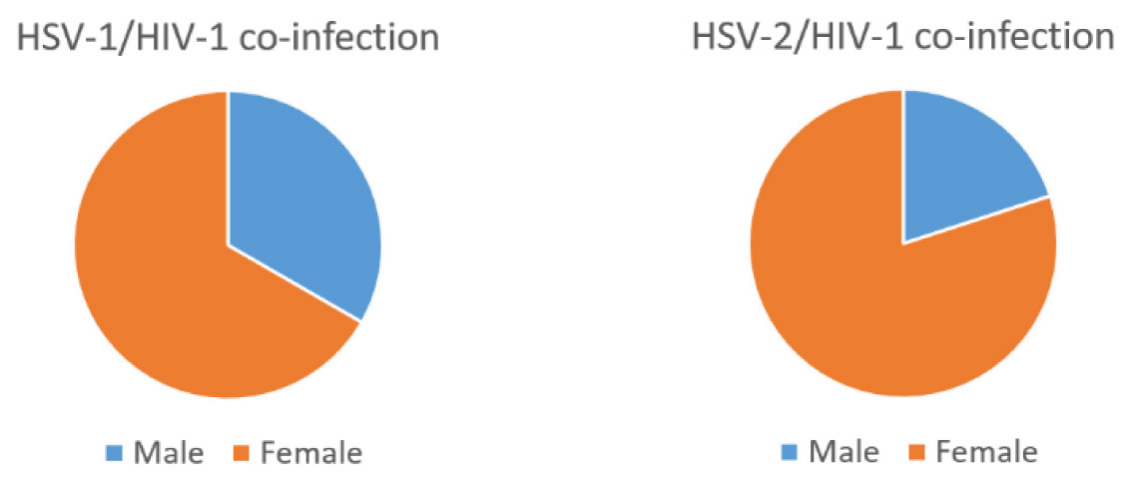
Gender categorization of PCR data of the HSV-2/HIV-1 and HSV-1/HIV-1 co-infected sera.

Furthermore, a significant proportion of the population, 6/44 (13.6%) males and 24/44 (54.5%) females were HSV-2/HIV-1 co-infected ( Figure 1).

Furthermore, PCR was also used to amplify the integrase gene of HIV-1 genome in the samples and the data showed that 36/44 (81.8%) samples that were positive for HIV-1 p24 using ELISA were also PCR positive.

**Figure 2.**
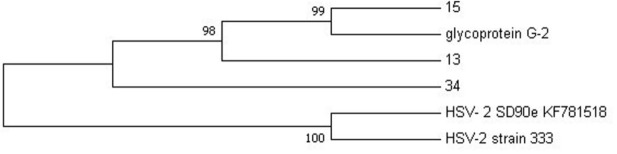
The evolutionary relationship between HSV-2 sequences (13, 15 and 34) and HSV-2 reference genomes was inferred using the maximum likelihood phylogeny method.

NGS was performed to confirm the genomes of HSV-2 and HIV-1 viral isolates that were detected using PCR. The samples that were sent for NGS sequencing were those that exhibited high titers with ELISA, that is, HSV-2 sequences 13, 15 and 34 and HIV-1 sequence G20. The sequenced data was compared with reference sequences obtained from NCBI that were selected based on the amplified targeted regions. A maximum likelihood phylogeny method of analysis was used with a bootstrap value of 1,000 replicates to generate evolutionary trees for HSV-2 and HIV-1, respectively. The phylogenetic analysis of HSV-2 sequence data showed that sequences 13 and 15 are more closely related to glycoprotein G2 reference strain than sequence 34, with 98–99% similarity to the G2 strain ( Figure 2).

**Figure 3.**
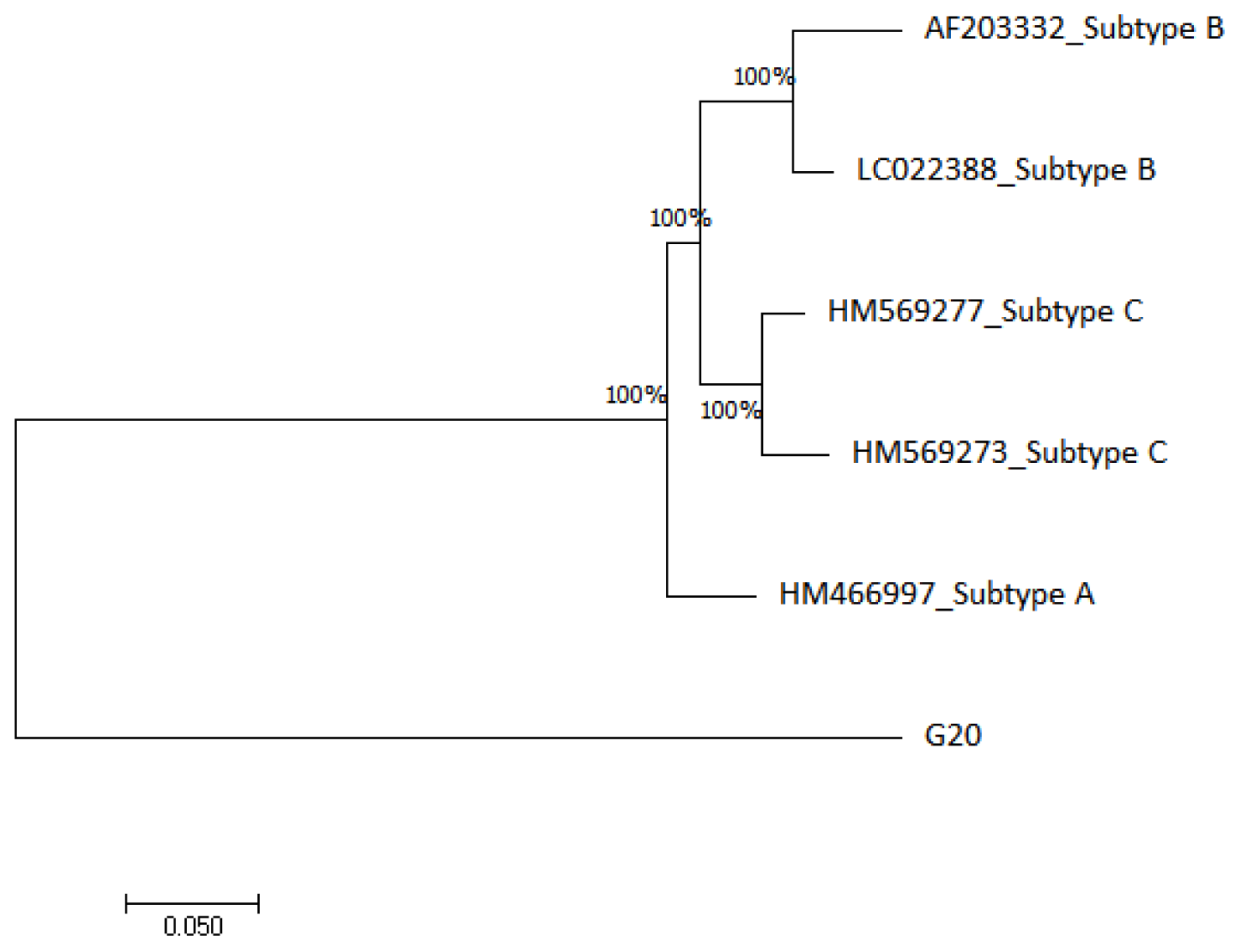
The evolutionary relationship of the HIV-1 sample (G20) with HIV-1 reference genomes from NCBI database was inferred using the maximum likelihood phylogeny method.

The HIV-1 phylogenetic tree in Figure 3 shows that G20 sequence is 100% closely related to subtype A (HM466997) and distantly related to subtype B (AF203332 and LC022388) and subtype C (HM569277 and HM569273) reference genomes.

The SPSS v25 statistical software was used to detect whether there is a relationship between age, HSV, and HIV-1. SPSS was also utilized to evaluate the association between HSV and HIV-1 positive samples ( Table 3). The two-tailed correlation test exhibited a statistical positive link between age and HSV-1 (0.366**) as shown in Table 3. HSV-2 and HIV-1 samples are also positively correlated (0.690**). In addition, an inverse or negative relationship between HSV-1 and HSV-2 (-0.463**) was detected.

**Table 3.** Correlation between age, HIV-1 ELISA positive samples, HSV-1 and HSV-2 PCR positive samples.

	AGE	HSV-1	HSV-2	HIV-1
**AGE**	1	0.366 **	-0.061	0.208
**HSV-1**	–	1	-0.463 **	-0.056
**HSV-2**	–	–	1	0.690 **
**HIV-1**	–	–	–	1

**Correlation is significant at 0.01 level (2-tailed)

The SPSS chi-square goodness-of-fit test was also used to assess association between HSV and HIV-1 positive samples. The data showed that there was no significant association between HSV-1 and HIV-1 (X ^2 ^(1) = 0.138, p>0.05). However, there was a strong statistical association between HSV-2 and HIV-1 (X ^2 ^(1) = 20.952, p< 0.05), as shown in Table 4.

**Table 4.** Association between HIV-1 ELISA positive samples and HSV-1 and HSV-2 PCR positive samples.

	HIV-1 ELISA			
	X ^2^ Value	Degree of freedom (df)	Asymptotic Significance (2-sided)	Interpretation
**HSV-1 PCR**	0.138	1	0.711	No significant association
**HSV-2 PCR**	20.952	1	0.000 *	Significant association

*Significant at 0.05 level (2-tailed).

## Discussion

There are insufficient data to recount the prevalence of HSV genotypes among HIV-1 infected individuals in the Republic of South Africa. This study provides insight into an existing interrelation between HSV and HIV-1 and the potential risk that one of the viruses may have on the other. Our focus was to determine the seroprevalence of HSV among HIV-1 sample cohort and further assess for possible co-infections (HSV-1/HIV-1 or HSV-2/HIV-1). In the study, 81.8% and 79.5% of the samples were positive for HIV-1 and HSV, respectively. Of the positive HSV samples, 85.7% were positive for HSV-2. Conversely, the most prevalent type of HSV (HSV-1) as recorded by previous studies ( Debrah *et al*., 2018; Looker & Garnett, 2005), was not highly prevalent in this study population (11.4%). This may be due to the small sample size as HSV-1 infection is often acquired during childhood and there were no childhood participants in the study (age range 1–10 years). Although, Debrah *et al.* used a larger sample cohort in their study, there was no record of childhood participants. Most of the high HSV-1 prevalence recorded was for participants aged 25–44 years. This low prevalence was also observed in a study conducted in the USA by Ayoub *et al*. (2019), where they examined the progression of HSV-1 epidemiology in the country. The study showed that more children will reach the age of sexual debut with no antibody protection against HSV-1. In addition, the low rate is attributable to the change in disease spread in the population since there are reduced viral HSV-1 antibodies at a very young age, a factor influencing HSV-2 acquisition.

Our study also revealed a higher rate of HSV-2/HIV-1 co-infection (13.6% males, 54.5% females) compared with HSV-1/HIV-1 (2.3% males, 4.5% females).Thus, one of the few observations drawn from this study was the relatively high prevalence of HSV-2 compared to HSV-1 in HIV-1 co-infected samples. The validity of the high prevalence of HSV-2/HIV-1 co-infection in this study is supported by previous studies ( Looker *et al.,* 2017; Patel *et al.,* 2012). The increase in HSV-2/HIV-1 co-infection is attributed to the route of viral transmission since both viruses share a similar route of transmission. Furthermore, it was also discovered that females were more susceptible to HSV-2 infection 30/35 (85.7%) than their male counterparts 4/35 (11.4%) in this population. This correlates with the findings of Pebody *et al.* (2004) and Smith & Robinson (2002), who reported that women are more at risk of acquiring HSV-2 infection compared with men. Similar findings were also observed by Celum *et al*. (2004) that more than half of the female population who are HIV-1 positive suffer from HSV-2 infection. This might be due to their early exposure to sexual relations than their male counterpart ( Schiffer *et al.*, 2014). Another probable explanation for the high prevalence of HSV in the female participant could be related to the anatomy of the female reproductive system. That is, the large surface area and the thin lining of the female reproductive system could facilitate HSV-2 entry ( Hladik & McElrath, 2008). Furthermore, the hydration and alkalinization of the mucus plug during ovulation in women, hinders the barrier function of the endocervical canal against pathogens hence, facilitating viral entry ( Hladik & McElrath, 2008). This is supported by the high number of HSV-2 and HIV-1 prevalence observed in the age group of 21–40 years, with only 3/44 (6.8%) HSV-1/HIV-1 co-infected samples. Of note is increased rate of co-infection with age, which correlates with the steady rise by age for HSV-2/HIV-1 co-infection, as recorded by Beydoun *et al.* (2010).

PCR was performed to confirm the positivity of the ELISA screened samples and differentiate the HSV samples into types 1 and 2. The HSV-2 results (85.7%) correlate with the high rate of HSV-2 infection in Africa. This was also observed in a similar study by Debrah *et al*. where they assessed the seroprevalence of HSV-1 and HSV-2 among women in Ghana and discovered that 78.4% of the study population are positive for HSV-2 ( Debrah *et al*., 2018). In addition, the prevalence of HSV-2 in this study is higher than HSV-2 prevalence in other African countries like Zimbabwe (68%) and Uganda (58%) ( Kurewa *et al.,* 2010; Nakku-Joloba *et al.,* 2014).

Phylogenetic analysis revealed that HSV-2 samples 13 and 15 from this study do not share the same ancestral lineage with a more virulent clinical isolate SD90e (accession number KF781518) from South Africa ( Newman *et al*., 2015) and the HSV-2 laboratory strain 333 (M15118). However, sample 34 is closely related to SD90e. Thus, the distant relation between SD90e reference genome and samples 13 and 15 may suggest geographical diversity in viral transmission within South Africa. This was also discovered in a study conducted by Newman *et al*. (2015), where they explored the geographical diversity between HSV-2 sequences and observed that HSV-2 sequences from Uganda are strongly related to the HSV-2 sequence from USA. Furthermore, a close relation of the two samples (G13 and G15) was observed with the less virulent glycoprotein G-2 strain originating from Scotland, which might be attributed to international migration. This was also observed by Szpara *et al*. (2014) where African strains isolated in Kenya were shown to cluster closely with Europe, North America, and Asian strains.

The HIV-1 phylogenetic analysis confirmed that the G20 sequence is an HIV-1 sequence however, it did not cluster with any of the subtype A, B or C reference sequences that were used. The G20 distant clustering may reflect another instance of international migration. For example, Lurie *et al*. (2003) demonstrated that migrants have a two-fold odd of contracting HIV than non-immigrants in South Africa. Similarly, migration from different geographical locations could influence viral transmission of different HIV-1 subtypes. This was demonstrated by von Wyl *et al.* (2011), where they revealed that 80% of non-B HIV-1 subtypes recorded in their study population originated outside the study region, Switzerland.

A previous study on the relationship between age and HSV-1 prevalence by Smith & Robinson (2002) reported that global HSV-1 prevalence increases with age. The statistical relationship between age, HSV and HIV-1 infection in our study revealed a similar significant relationship between age and HSV-1 (p = 0.366*). Similarly, we discovered a discreet relationship between age, HSV-2 and HIV-1. However, HSV-1 showed an inverse correlation with HSV-2 (p = -0.463 ^**^). This may suggest that an increase in HSV-2 prevalence in the population will result in a decline in HSV-1 as supported by Ayoub *et al*. (2019). It was also discovered that a robust significant relationship exists between HSV-2 and HIV-1 (p =0.690 ^**^) suggesting that a steady rise in HSV-2 contributes to an increase in HIV-1 infection in the population. A probable reason for this relationship is that the viruses share a similar route of entry and the impact of one is significant on the other as seen in the micro-ulceration of the genitalia in HSV-2 patients, which provides a port of entry for HIV-1 ( Sheth *et al*., 2008).

The association between HIV-1 and HSV was analysed using chi-square goodness-of-fit test and it was discovered that no significant association exist between HSV-1 and HIV-1 (X ^2 ^(1) = 0.138, p >0.05) but a strong statistical association was found between HSV-2 and HIV-1 (X ^2 ^(1) = 20.952, p <0.05). Although, there has been contrasting opinions on the association of HSV-2 and HIV-1, the current study is consistent with Sudenga *et al.* (2012) and Omori & Abu-Raddad (2017) who suggested that the infection of one virus may fuel the transmission of the other. In their studies, Sudenga *et al.* (2012) observed that HIV-1 positive individuals with higher CD4+ counts at baseline and those with lower viral load were associated with HSV-2 acquisition, while Omori & Abu-Raddad (2017) used the sexual network determinants as components to determine the prevalence of HIV-1/HSV-2. They deduced that HIV is an agent of HSV-2 transmission in the population. However, another study led by Kouyoumjian *et al.* (2018) discovered a robust association between HSV-2 and HIV-1, with HSV-2 prevalence being consistently higher than HIV-1 in the global population. The data from this study suggests that contracting one virus (either HSV-2 or HIV-1) will influence the acquisition of the other. This highlights the importance for data collation on HSV-2 and HIV-1 infected persons in South Africa.

### Limitations

The study is a retrospective study with the aim to determine the prevalence of HSV-DNA in HIV-1 sera. However, there were certain limitations. Primarily, the study population was small when compared to the size of the general population. In addition, the sera samples were collected exclusively from North-West and KwaZulu-Natal Provinces, poses a challenge to generalize the outcomes of the study to the South African populace.

## Conclusion

This study revealed a clustering variation of HSV-2 sequences. The clustering differentiated the sequences from the prominent sequence (SD90e) found in South Africa, which suggests diversity in the transmission of the virus. Similarly, a different HIV-1 sub-type was isolated from the study population, different from other HIV-1 sub-type C isolates in South Africa. Despite the small sample size, a high prevalence of HSV-2/HIV-1 co-infection (68.2%) was recorded which shows that a positive association exists between HSV-2 and HIV-1, suggesting that an increase in one of the viruses may influence the spread of the other. Thus, acknowledging that a relationship exists between these two viruses, and to identify how the transmission of one could affect the other, requires a larger cohort that is well described with longitudinal measurements of HSV-1, HSV-2, and HIV-1 as well as measurements of potential confounders such as condom use, partner change and other sexually transmitted diseases.

## Data availability

### Underlying data

SRA: gG sequencing of HSV-2 (sample G15), Accession number SRX9590097: https://www.ncbi.nlm.nih.gov/sra/?term=SRX9590097

SRA: gG sequencing of HSV-2 (sample G34), Accession number SRX9590139: https://www.ncbi.nlm.nih.gov/sra/?term=SRX9590139

SRA: gG sequence (sample G13), Accession number SRX9590098: https://www.ncbi.nlm.nih.gov/sra/?term=SRX9590098

SRA: HIV-1 sequencing targeting integrase region (sample G20), Accession number SRX9531105: https://www.ncbi.nlm.nih.gov/sra/?term=SRX9531105

Dryad: HSV AND HIV RAW DATA, https://doi.org/10.5061/dryad.zs7h44j7g ( Obisesan *et al.,* 2020).

This project contains the following underlying data:

- CSV spreadsheet containing demographics and ELISA and PCR results for all 44 sera samples.

### Extended data

Dryad: F1000 SUPPLEMENTARY FILE, https://doi.org/10.5061/dryad.zs7h44j7g ( Obisesan *et al.,* 2020).

This project contains the following extended data:

- Table A: Primers for the detection of HSV-1, HSV-2 and HIV-1.
- Table B: PCR reaction mixture and thermocycling conditions for HSV-1 and -2.
- Table C: Thermocycling conditions for HIV-1 PCR.
- Figure A: ELISA results of HSV as recorded on the microplate reader.
- Figure B: Age variation and the frequency distribution of HSV-1 in the study population
- Figure C: Age variation and the frequency distribution of HSV-2 in the study population

Data are available under the terms of the Creative Commons Zero "No rights reserved" data waiver (CC0 1.0 Public domain dedication).
