# Supplementary material for: Seroprevalence and characterisation of herpes simplex virus from human immunodeficiency virus in samples collected from two provinces in South Africa: a retrospective study
Source: F1000Res. 2021 Nov 17;10:105. Originally published 2021 Feb 11. [Version 4] doi: 10.12688/f1000research.28105.4 (PMC8591517; doi:10.12688/f1000research.28105.4)
Supplement: Peer review history

# Peer review: Reviewer response for version 3

**DOI:** 10.5256/f1000research.78435.r97114

**Published:** 2021-11-11

**Authors:** Bongiwe Ndlovu

**Affiliations:** 1 HIV Pathogenesis Programme, University of KwaZulu-Natal, Durban, South Africa

I am happy with the revised manuscript. Although, a few points should be addressed before the study is indexed. Conclusions should be revised, the authors mentioned that "There is a significant relationship between HSV-2 and HIV-1 in the study population". Are they referring to a positive association? This should be clarified.

 In sample collection criteria and study population, the authors mentioned that "six samples were lysed and were excluded". This is confusing, are they referring to poor sample quality? This should also be clarified. Which stop solution did they use to terminate ELISA experiments?

Is the work clearly and accurately presented and does it cite the current literature?

Partly

If applicable, is the statistical analysis and its interpretation appropriate?

I cannot comment. A qualified statistician is required.

Are all the source data underlying the results available to ensure full reproducibility?

Yes

Is the study design appropriate and is the work technically sound?

Yes

Are the conclusions drawn adequately supported by the results?

Partly

Are sufficient details of methods and analysis provided to allow replication by others?

Partly

Reviewer Expertise:

HIV Immunology

I confirm that I have read this submission and believe that I have an appropriate level of expertise to confirm that it is of an acceptable scientific standard.

# Response

**Published:** 2021-11-15

**Authors:** oluwafemi obisesan

**Affiliations:** North-West University, South Africa

Dear Editors,

 We would like to thank you for considering our manuscript (28105) entitled “Seroprevalence and characterisation of herpes simplex virus from human immunodeficiency virus in samples collected from two provinces in South Africa: a retrospective study” for publication in your reputable journal. We also appreciate the reviewers’ efforts, comments, and concerns in making the manuscript better. We have gone through the reviewer’s comments and edited the manuscript accordingly in the revised manuscript. The inputs made in response to the reviewer’s comments are listed below.

**Reviewer’s comments**:

1. Conclusions should be revised, the authors mentioned that "There is a significant relationship between HSV-2 and HIV-1 in the study population". Are they referring to a positive association? This should be clarified.

***Response*** *: Yes, we are referring to a positive association. We have made the necessary clarification to the conclusion section in the abstract.*

  

1. In sample collection criteria and study population, the authors mentioned that "six samples were lysed and were excluded". This is confusing, are they referring to poor sample quality? This should also be clarified.

***Response*** *: We are referring to the quality of the six samples. The samples were lysed and were excluded from the experiment. This is now clarified in the methods section on page 4.*

  

1. Which stop solution did they use to terminate ELISA experiments?

***Response*** *: The Bio-Rad kit came with its own stop solution of 0.3M sulphuric acid. This was the reason it was not mentioned in the previous version. It is now stated in methods section on page 4.*

# Peer review: Reviewer response for version 3

**DOI:** 10.5256/f1000research.78435.r97115

**Published:** 2021-10-18

**Authors:** Debrah Oksana

**Affiliations:** 1 Department of Molecular Medicine, Kwame Nkrumah University of Science and Technology, Kumasi, Ghana

The manuscript has been improved during the last revision. I commend the authors for carefully addressing the reviewers' feedback. The manuscript can be accepted for indexing.

Is the work clearly and accurately presented and does it cite the current literature?

Partly

If applicable, is the statistical analysis and its interpretation appropriate?

Partly

Are all the source data underlying the results available to ensure full reproducibility?

Yes

Is the study design appropriate and is the work technically sound?

Partly

Are the conclusions drawn adequately supported by the results?

Partly

Are sufficient details of methods and analysis provided to allow replication by others?

Partly

Reviewer Expertise:

Infectious diseases of public health concern, medical biochemistry, chemical pathology

I confirm that I have read this submission and believe that I have an appropriate level of expertise to confirm that it is of an acceptable scientific standard.

## References

[1] : Seroprevalence and characterisation of herpes simplex virus from human immunodeficiency virus in samples collected from two provinces in South Africa: a retrospective study. *F1000Research* .2021;10: 10.12688/f1000research.28105.3 10.12688/f1000research.28105.3 PMC859151734853674

# Peer review: Reviewer response for version 2

**DOI:** 10.5256/f1000research.77515.r95089

**Published:** 2021-10-01

**Authors:** Debrah Oksana

**Affiliations:** 1 Department of Molecular Medicine, Kwame Nkrumah University of Science and Technology, Kumasi, Ghana

**General Comments:**

 The manuscript has been improved during the revision.

 For the results section, I have some suggestions:

1. Due to small sample size, author should categorize age on three categories only: >20 years, 21-40 years & 41-60 years.
2. Table 1 & Table 2: kindly specify that the data is number of positive/ negative cases/ male & female. You can put near the parameter “N”.

Conclusion section should be revised. The sample size is very small to make the statement such as “…HSV is highly prevalent, and women are mainly affected. Moreover, HSV-2 infection in the study cohort was robustly associated with HIV-1”. Similarly review the conclusion in Abstract.

 Limitation section should be revised. Sample size is very small to make statement such as “The study contributed to literature by exploring a broad group of participants between 11 to 60 years”.

 It is suggested that authors focused in their conclusion more on the sequencing analysis of the viruses, and present the prevalence of co-infection as side data, due to small sample size.

**Comments:** Recommend for indexing after revisions are made.

Is the work clearly and accurately presented and does it cite the current literature?

Partly

If applicable, is the statistical analysis and its interpretation appropriate?

Partly

Are all the source data underlying the results available to ensure full reproducibility?

Yes

Is the study design appropriate and is the work technically sound?

Partly

Are the conclusions drawn adequately supported by the results?

Partly

Are sufficient details of methods and analysis provided to allow replication by others?

Partly

Reviewer Expertise:

Infectious diseases of public health concern, medical biochemistry, chemical pathology

I confirm that I have read this submission and believe that I have an appropriate level of expertise to confirm that it is of an acceptable scientific standard, however I have significant reservations, as outlined above.

# Response

**Published:** 2021-10-12

**Authors:** oluwafemi obisesan

**Affiliations:** North-West University, South Africa

Dear Reviewer,

 We appreciate the efforts, comments, and concerns in making the manuscript better. We have gone through the comments and edited the manuscript accordingly in the revised manuscript. The inputs made in response to your comments are listed below.

**General comments**:

1. Due to the small sample size, author should categorize age on three categories only: <20 years, 21-40 years & 41-60 years.

***Response*** *: We have categorized the age as suggested in Tables 1 & 2 on pages 6 and 7 of the revised manuscript.*

1. Table 1 & Table 2: kindly specify that the data is number of positive/ negative cases/ male & female. You can put near the parameter “N”.

***Response*** *: We have made clear demarcations of positive and negative HIV-1 and HSV infections in Table 1. The same has been done for Table 2 to differentiate HSV-1 and HSV-2 PCR positive males and females.*

1. Conclusion section should be revised. The sample size is very small to make the statement such as “…HSV is highly prevalent, and women are mainly affected. Moreover, HSV-2 infection in the study cohort was robustly associated with HIV-1”. Similarly, review the conclusion in Abstract

***Response*** *: The conclusion section has been revised, focusing more on the sequence analysis of the viruses as outlined on page 12.*

  

1. Limitation section should be revised. Sample size is very small to make statement such as “The study contributed to literature by exploring a broad group of participants between 11-60 years

***Response*** *: Thank you for noting this. We have excluded the statement from the limitation section on page 12.*

# Peer review: Reviewer response for version 1

**DOI:** 10.5256/f1000research.31089.r80471

**Published:** 2021-04-14

**Authors:** Bongiwe Ndlovu

**Affiliations:** 1 HIV Pathogenesis Programme, University of KwaZulu-Natal, Durban, South Africa

**Study Title: **The title of the paper should be revised, it is unclear. The location of samples collected should be removed from the title and properly explained in the Methods section. 

**The introduction: **The authors should restructure the sentences. Some sentences are long and they have typographical errors. Some statements are inaccurate, for example: they mentioned that transmission is asymptomatic. 

**Methods:** The methods section should be revised. The authors should indicate the type of samples used for the study (blood, plasma, or serum), the country of origin, and the hospitals/ institutions where the samples were collected. They should also define convenience sampling. 

**Laboratory Analysis:** The authors should restructure the sentences. They also used abbreviations like EIA & PCR without describing them first/ writing in full.

**ELISA **method was not properly described. Which HIV-1/ HSV proteins did they use to measure antibody responses? 

 The authors also did not specify which genes of interest and primers were used to amplify the HIV-1 genome with nested PCR. This should be clearly explained and referred to supplementary information.

**Sequencing:** It is not clear why G13, G15, G34 were sequenced with HSV-2 primers while G20 was amplified with HIV-1 primers. 

**Results:**

- Did you measure p24 antibody titers?
- Figure 1 is confusing, the authors used too many colors. They should consider simplifying the figure by showing a bar graph with positive and negative responses for each age group. The figure legend is too brief.
- The authors did HSV1/2 PCR. However, the results are not shown in the article. The bar graphs are only shown in the supplementary information. These should be simplified and added as figure 2 of the manuscript. Where is the figure that shows HIV-1/HSV co-infections? These can be summarized clearly in a pie chart.
- The interpretation of the sequencing data needs improvement. Which HIV-1 subtypes are HIV LC201873, 4533/11 and KU609428? Are they South African strains? What was the aim of selecting these strains? 
- HIV-1 LC022388 and 3793/15 KU609388) were used in constructing the phylogenetic tree. However, it is not clear why these strains were selected for analysis. 
- The relationships between age and HSV-1, HSV-2, and HIV-1 or HSV-1 and HSV-2 are not clearly explained. Are they positive or negative associations? 

**Discussion: **

- The authors mentioned that HSV-1 is the most prevalent type of HSV as recorded by previous studies. However, they did not cite those studies. 
- In this study, they only found 4 patients with HSV-1. They should explain the differences between these studies that could lead to this difference in the findings.
- The differences in HSV prevalence between males and females, could be due to their anatomy? It is unclear whether the studies conducted by Cunningham 2006 and Ayoub 2019 were done on South African participants or not.

**Conclusion: **The conclusion should be revised. The study was done on a small sample size.

Is the work clearly and accurately presented and does it cite the current literature?

Partly

If applicable, is the statistical analysis and its interpretation appropriate?

I cannot comment. A qualified statistician is required.

Are all the source data underlying the results available to ensure full reproducibility?

Yes

Is the study design appropriate and is the work technically sound?

Yes

Are the conclusions drawn adequately supported by the results?

Partly

Are sufficient details of methods and analysis provided to allow replication by others?

Partly

Reviewer Expertise:

HIV Immunology

I confirm that I have read this submission and believe that I have an appropriate level of expertise to confirm that it is of an acceptable scientific standard, however I have significant reservations, as outlined above.

# Peer review: Reviewer response for version 1

**DOI:** 10.5256/f1000research.31089.r79452

**Published:** 2021-03-03

**Authors:** Debrah Oksana

**Affiliations:** 1 Department of Molecular Medicine, Kwame Nkrumah University of Science and Technology, Kumasi, Ghana

**General comments:**

1. Majority of the sentences can be restructured for better representation and understanding of information.
2. The text contains some grammatical and punctuation errors, they should be corrected before indexing.
3. It is suggested that the authors focus more on the sequencing analysis of the viruses, and present the prevalence of co-infection as side data, due to small sample size.

**Specific comments:**

 Below are more specific comments by section:

1. **Title: **The title of the article should be revised to make it more specific. For example, the country where the study took place should be included. This is an international journal and it is important to know from the title where these provinces can be found.
2. **Abstract: **It is advised to revise the structure of the “Abstract” to improve the quality of the paper (or example, background, conclusion etc.).
3. **Introduction: **Some sentences are very long and can be restructured for better representation and understanding of information (for example, first and second sentence, etc.). Objectives can be better expressed.
4. **Methodology**: Generally, this section can be improved. The details can be added to some laboratory analysis, even though the publication was done and the authors used the manufacturer’s instructions.
   
  - **Sample collection criteria and study population**: this section needs clarification. According to the authors, there was a retrospective study, but the methodology states that the sample was collected from the patients using convince sampling approach. Where later it was mentioned that the sample was collected blindly from the facilities. Please, kindly write in details the sampling collection approach so that it would be easy to understand. Secondly, why were different numbers of sera samples collected from two provinces (20 sera samples from North-West and 24 from KwaZulu-Natal Provinces)? It would be appropriate to explain the sampling approach that was used.
  - **Laboratory analysis**: the structure of this paragraph can be improved.
  - **ELISA**: It was mentioned that the kits used in research were not specific. This statement needs some clarification, is it applied for HSV test kit or for HIV-1 test kit also?
5. **Results**:

- Table: Presentation can be improved (for example, in table 1 there was no data for age group less than 10 years and etc.).
- Table 1 & figure 1 represent the same data. One should be removed, as it is a duplication of data representation.
- It was mentioned in the methodology section, that the PCR was used to confirm the non-specific ELISA method even for HIV-1. There is no data in the result section. Please, kindly include this data.
- Some information on result section should be sent to methodology section.

 

- **Discussion**: It is very difficult to draw the conclusion on the co-infection prevalence of HSV type 1 and type 2 and HIV-1, due to the small sample size and small co-infection in the study population.
- **References**: Some references in the text were wrongly introduced, for example, Debrah *et al.*, 2018 was cited to present prevalence of HSV in Gambia, whereas Debrah and colleagues did the research in Ghanaian population.
- **Limitation**: The main limitation of the study is small sample size. This section mentioned that the age of study participants is from 14-49 years, whereas the results (table 1) introduce the data up to 60 years.
- **Conclusion**: It is very difficult to draw the conclusions with such small sample size. The authors took a risk to make such conclusions.

**Conclusion: **major revision is needed.

Is the work clearly and accurately presented and does it cite the current literature?

Partly

If applicable, is the statistical analysis and its interpretation appropriate?

Partly

Are all the source data underlying the results available to ensure full reproducibility?

Yes

Is the study design appropriate and is the work technically sound?

Partly

Are the conclusions drawn adequately supported by the results?

Partly

Are sufficient details of methods and analysis provided to allow replication by others?

Partly

Reviewer Expertise:

Infectious diseases of public health concern, medical biochemistry, chemical pathology

I confirm that I have read this submission and believe that I have an appropriate level of expertise to confirm that it is of an acceptable scientific standard, however I have significant reservations, as outlined above.
